# Regulation of the G1/S Transition in Hepatocytes: Involvement of the Cyclin-Dependent Kinase Cdk1 in the DNA Replication

**DOI:** 10.1155/2012/689324

**Published:** 2012-10-03

**Authors:** Anne Corlu, Pascal Loyer

**Affiliations:** Inserm UMR S 991, Foie Métabolismes et Cancer, Université de Rennes 1, Hôpital Pontchaillou, 35033 Rennes Cedex, France

## Abstract

A singular feature of adult differentiated hepatocytes is their capacity to proliferate allowing liver regeneration. This review emphasizes the literature published over the last 20 years that established the most important pathways regulating the hepatocyte cell cycle. Our article also aimed at illustrating that many discoveries in this field benefited from the combined use of *in vivo* models of liver regeneration and *in vitro* models of primary cultures of human and rodent hepatocytes. Using these models, our laboratory has contributed to decipher the different steps of the progression into the G1 phase and the commitment to S phase of proliferating hepatocytes. We identified the mitogen dependent restriction point located at the two-thirds of the G1 phase and the concomitant expression and activation of both Cdk1 and Cdk2 at the G1/S transition. Furthermore, we demonstrated that these two Cdks contribute to the DNA replication. Finally, we provided strong evidences that Cdk1 expression and activation is correlated to extracellular matrix degradation upon stimulation by the pro-inflammatory cytokine TNF**α** leading to the identification of a new signaling pathway regulating Cdk1 expression at the G1/S transition. It also further confirms the well-orchestrated regulation of liver regeneration via multiple extracellular signals and pathways.

## 1. Introduction

The cell cycle is highly conserved cellular process allowing a cell to divide in two identical daughter cells. Although mammalian cells show a higher degree of complexity, the molecular pathways controlling the progression throughout the cell cycle and both DNA replication and mitosis are relatively well conserved among eukaryotic cells [1]. The most conserved pathways of the cell cycle are probably DNA replication and major check-points for DNA integrity and mitosis. In contrast, more specific pathways control the transition from quiescence to DNA replication in eukaryotic organisms. In mammalian cells, specific combinations of extracellular signal stimuli induce the exit from quiescence, progression throughout G1 phase, and commitment to DNA replication. Proliferation stimuli include a vast superfamily of growth factors and cytokines activating downstream intracellular signaling pathways mainly through a cascade of phosphorylation and dephosphorylation events that ultimately triggers changes in gene expression in order to induce the proteins required for duplication of cellular components including DNA and the subsequent mitosis [2]. Among these protein kinases, the sequential activation of the cyclin-dependent kinases (Cdks) has been extensively characterized and plays a crucial role in regulating the entry into and progression through the cell cycle [3]. 

The discovery of the first Cdk, Cdk1 initially named cdc2 in yeast, has opened a large field of research leading to the identification of many cell cycle regulators and the pathways they are involved into. The first studies regarding the cell cycle regulation were conducted using cell models such as yeasts and oocytes from amphibians and marine organisms that synchronously progress throughout the different phases of the cell cycle in order to analyze expression and activation of regulators at each step of the cell cycle. From the mid-1970s to the late 1980s, the burst of data obtained in these eukaryotic cells leads to the identification of major cell cycle regulators including the cyclins [4] and their catalytic subunit partners the Cdks [3]. Mammalian homologs of these cell cycle regulators were subsequently isolated and by the mid-1990s a network of Cdk/cyclin complexes emerged opening a complete new field in cancer research since many of these cell cycle regulators are altered during oncogenesis and/or are potential therapeutic targets for cancer treatments [5].

*In vivo*, cell renewal is mainly achieved through the proliferation of adult stem and progenitor cells that proliferate actively although these cells can probably arrest in G0 before additional rounds of division or entering a program of differentiation. Because progenitor cells are rare cells and cannot be easily purified, there are few data regarding cell cycle regulation in these cell types. There are, however, adult differentiated cell types that remain arrested in G0, which can reenter the cell cycle for several rounds of division upon appropriate proliferation stimuli including lymphocytes [6] and fibroblasts [2] which can be isolated relatively easily from blood or skin, respectively, plated in culture and used for cell cycle studies. Although these cell types are suitable models for conducting cell cycle studies, there have been a limited number of publications reporting cell cycle data using lymphocytes and mainly because these primary cells need to be renewed for each experiment. The most widely used cell models in the field of cell cycle regulation are the immortalized or transformed cell lines artificially synchronized by drug treatments arresting the cells in G1/S or G2/M transitions and the primary fibroblasts arrested by serum starvation in a G0-like state. Although the scoop of this paper is to focus on the progression in late G1 and the G1/S transition, it is important to point out that the comparison between these *in vitro* models of G0-like or early G1 arrest and *in vivo* G0 arrested cells was poorly documented for many years. However, recent reports evinced differences between “arrested” cells in various conditions [7, 8]. For instance, the serum starvation of fibroblasts plated at low density obviously provides an experimental condition completely different from G0-arrested cells *in vivo*, which stop dividing for other reasons than the lack of growth factors or nutrients. Nevertheless, these *in vitro* synchronized mammalian cells provided powerful models to investigate cell cycle in mammalian cells and allowed to collect crucial data on the progression from early G1 to the commitment to DNA synthesis. 

In mammals, synchronized cell proliferation *in vivo* is restricted to very few cell types among which proliferation of hepatocytes during liver regeneration following partial hepatectomy has probably been the most used model. In this paper, we will focus on the peculiar regulation of the Cdk1 expression and activation during the hepatocyte cell cycle.

## 2. *In Vivo* and *In Vitro* Models of Synchronized Hepatocyte Proliferation

**Figure 1:**
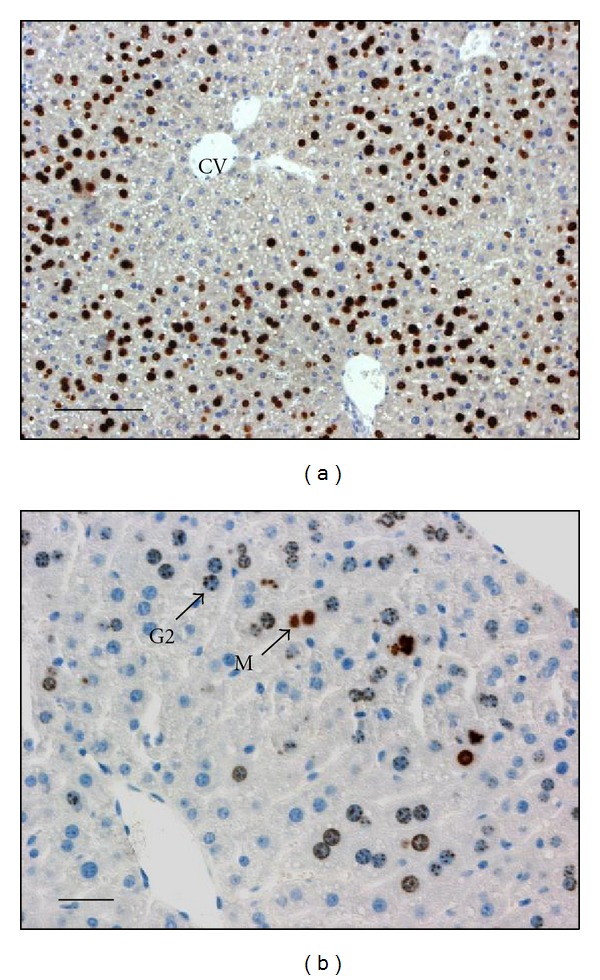
Sections of mouse liver evidencing detection of DNA replication and G2 phase. Mice were hepatectomized, injected at 46 hours after hepatectomy with BrdU, and killed 2 hours later (at 48 h). Livers were fixed for histological studies and detection of BrdU to visualize hepatocytes replicating DNA (a) or phosphohistone H3 (b) to detect cells in G2 phase. (a) This low magnification picture shows the detection of BrdU positive cells replicating DNA, illustrating that replicating hepatocytes are initially localized in the vicinity of the portal vein while around centrolobular veins (CV) only few hepatocytes replicate DNA at 48 h. (b) A higher magnification picture shows nuclei of hepatocytes reaching G2 phase (detection of phosphohistone H3 positive cells with punctuated nuclear signal: G2 and mitosis (M). Bars: 100 *μ*m.

In contrast to other regenerating tissue, the liver regeneration process involves massive proliferation of differentiated hepatocytes in the remnant tissue (Figure 1). The liver regeneration is triggered experimentally by liver resection or by injection of hepatotoxic agent leading to cell death either by necrosis or apoptosis such as the thioacetamide [9] or CCl4 [10]. However, the most commonly used model of liver regeneration is the partial hepatectomy in rat or mouse. After 2/3 hepatectomy, liver regeneration begins with a first synchronous wave of hepatocyte proliferation, followed by sequential proliferation of biliary, kupffer, and endothelial cells [11, 12]. Proliferation of mature hepatocyte first occurs within the parenchyma in the vicinity of the portal triads and proceeds to the pericentral area close to the centolobular veins [13] (Figure 1). The unique ability of differentiated hepatic cells to exit from quiescence and reenter the cell cycle after a tissue loss has aroused numerous studies to identify exogenous factors triggering the liver regeneration and regulators of hepatocyte cell cycle progression. Both *in vivo* and *in vitro* models have been extensively studied for identifications of the extracellular stimuli regulating cell cycle of mature hepatocytes and downstream signaling pathways. 

Using *in vivo *models, Molten and Bucher have shown that circulating growth factors present in the serum of hepatectomized rats induce hepatocyte replication in parabiosed nonhepatectomized animals [14]. Using primary culture of rat hepatocytes, HGF, TGF*α*, EGF, heparin-binding EGF-like growth factor (HB-EGF), and amphiregulin have been identified as potent hepatocyte growth factors [12]. However, the injection in rat of these growth factors does not induce massive hepatocyte DNA replication since normal hepatocytes *in vivo* are not able to respond to mitogenic signal without priming events allowing hepatocytes to become “sensitive” to growth factors. The proinflammatory cytokines TNF*α* and IL-6 are the early stimulus during the liver regeneration allowing the exit of hepatocytes from quiescence and the priming of hepatocytes [15, 16]. Rapid induction of urokinase activity and urokinase receptor expression appeared within 5 min followed within 30 min by a rapid activation of NFkB and STAT3. These transcription factors participate to the induction of a subset of genes called “immediate early genes” including c-fos and c-Jun leading to an increase in AP1 activity.

Then high levels of HGF are found in plasma around two hours after PH. This initiation phase controlled by proinflammatory cytokines thus results in the G0/G1 transition and early G1 progression allowing hepatocytes to become sensitive to growth factors and competent for commitment to DNA replication. Therefore, the complex regenerating process is now divided in three distinct phases: the initiation, proliferation, and termination steps. In rat and, to a lesser extent, in mouse the first wave of hepatocyte proliferation following partial hepatectomy (PH) is synchronous. In both rat and mouse, within less than 15 minutes after the PH, hepatocytes exit quiescence and enter in G1-phase [17]. The timing of DNA replication and mitosis is however different between the two species. The peak of DNA synthesis is observed at 22–24 h in rat followed by a peak of mitosis at 28–30 h [18–22] while DNA replication occurs nearly 24 h later in mice. Seven days later, the liver has recovered nearly 70% of its initial mass. 

Isolation of hepatocytes from rodent and human liver and establishment of *in vitro* culture systems have provided powerful experimental *in vitro* models to identify extracellular signals and to study intracellular signaling pathways regulating differentiation and controlling the ratio between proliferation and apoptosis in liver. Enzymatic liver dissociation triggers G0/G1 transition of quiescent hepatocytes, which progress up to and arrest in mid-G1 phase in absence of growth factors in primary culture [23, 24]. It has been proposed that rupture of cell-cell interactions [23] and induction of oxidative stress [25] or proinflammatory response [26] during liver dissociation could be responsible of hepatocytes reentry into the cell cycle, mimicking the effect of proinflammatory cytokines TNF*α* and IL6 which control the G0/G1 transition *in vivo* during liver regeneration [11, 27]. In agreement, we demonstrated that TNF*α* was released into the perfusion buffers during the *in situ* procedure of hepatocyte isolation by collagenase dissociation of the rat and mouse liver with various amounts ranging from 100 to 500 pg/mL (Corlu A and Loyer P, unpublished data).

**Figure 2:**
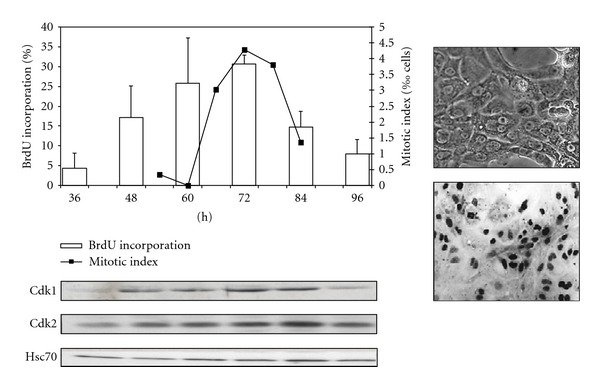
Proliferation of rat hepatocytes in pure culture. Isolated rat hepatocytes (right, picture in phase contrast) seeded at low density (<5.10^4^ cells/cm^2^) and stimulated with EGF (25 ng/mL) commit to DNA synthesis (BrdU incorporation, histogram and *in situ* immunodetection of BrdU positive cells) and complete the cell cycle (mitotic index, right axis). Western blotting of Cdk1 and Cdk2 and the loading control Hsc70.

In pure culture of hepatocytes, expression of liver specific functions progressively decreases and apoptosis eventually occurs within a week through the activation of caspases 3, 8, and 9 in hepatocytes [28–30]. Nevertheless, this *in vitro* culture model has been very useful to identify survival factors and mitogens based on their ability to induce DNA replication. In pure culture of rat hepatocytes, addition of 25 ng/mL of EGF in the culture induces a robust and partially synchronized DNA replication followed by the mitosis (Figure 2). Using this synchronous primary model, our laboratory and others investigated cell cycle regulation Cdk/cyclin expressions and activations [12, 31–33]. 

**Figure 3:**
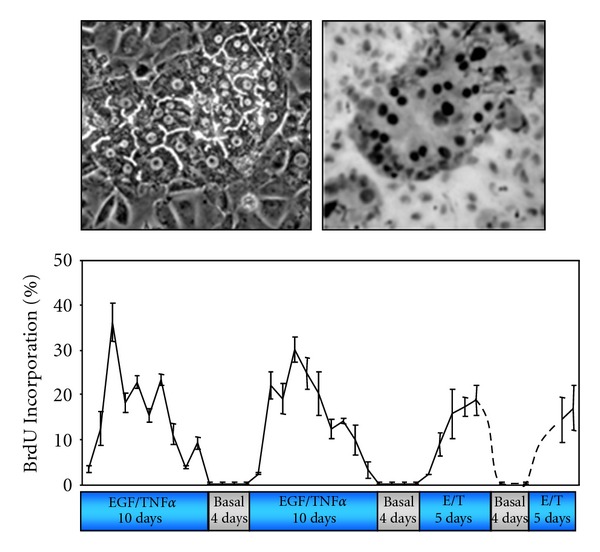
Proliferation of rat hepatocytes in coculture. Upper left: phase contrast picture of a colony of rat hepatocytes surrounded by Rat Liver Epithelia Cells (RLECs). Upper right: indirect immunodetection of BrdU positive hepatocytes evidencing DNA replication within the hepatocyte colony. Chart: multiple waves of replication in hepatocytes maintained in coculture over 52 days. Four periods of stimulation using EGF and TNF*α* (E/T) were separated by culturing the cells in basal medium lacking the mitogenic cocktail. The BrdU was incorporated for 24 h and at each time point triplicate cultures were fixed and stained for BrdU detection.

More recently, we used a coculture model associating rat hepatocytes with rat liver epithelial cells (RLEC also called BEC for biliary epithelial cells), in which heterotypic cell-cell contacts are restored and a spontaneous early production and deposition of extracellular matrix are observed [34–36]. This coculture model (Figure 3) compared to the pure culture of hepatocytes exhibits numerous advantages: adult hepatocytes remain highly differentiated for several weeks [37] and are unable to proliferate under EGF or HGF stimulation alone as in liver tissue [38]. Therefore, based on the data obtained *in vivo*, we successfully designed a stimulation procedure allowing multiple hepatocyte division cycles without loss of differentiation [39]. In this coculture system, differentiated and quiescent hepatocytes are able to proliferate under costimulation with TNF*α* and EGF or HGF.

This co-stimulation with TNF*α* and EGF leads to proliferation of nearly all the hepatocytes over a week [39]. Three days after TNF*α*/EGF or TNF*α*/HGF stimulation, at least 35% of hepatocytes divide whereas no DNA synthesis is observed in presence of HGF or EGF alone. Both mono- and binuclear hepatocytes progressed up to mitosis and cytokinesis allowing the significant expansion of hepatocyte colonies. These results are in agreement with *in vivo* experiments, in which coinjection of TNF*α* and growth factors induced hepatocyte proliferation in absence of partial hepatectomy [15]. Moreover, TNF*α* alone did not induce hepatocyte proliferation in coculture as observed *in vivo* [15] and in long-term DMSO cultures [40]. Remarkably, hepatocytes gradually stop synthesizing DNA even under prolonged TNF*α*/EGF stimulation. We demonstrated that a cell cycle arrest following the first wave of divisions is essential for inducing a second round of proliferation. Although cells do not proliferate in a synchronous manner in this coculture model, this *in vitro* cell system mimics the behavior of the hepatocytes in the whole liver and was used to investigate the involvement of cell-cell and cell-matrix interactions in the regulation of the hepatocyte cell cycle. 

## 3. The G1 Phase and the Mitogen-Dependent Cell Cycle Progression

**Figure 4:**
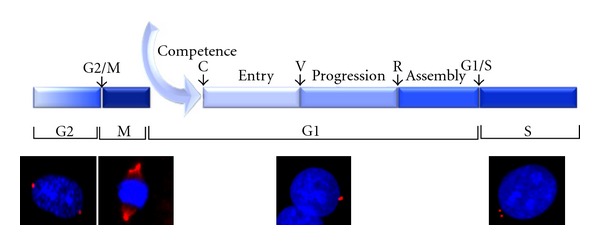
Progression through the G1 phase is divided into several subphases. Photographs illustrate detection of cells in G2, M, G1, and S phases: the cells were stained with DAPI (DNA) and indirect immunofluorescence detection of *γ*-tubulin in centrioles was used to discriminate between cells in G1 phase (a single centrosome), S phase (two centrosomes side by side), G2 (two centrioles on each side of the nucleus), and M (centrioles pulling apart the chromosomes). Four steps were identified during the G1 phase of the cell cycle: competence, entry, progression, and assembly.

Nearly three decades ago, the *in vitro* synchronized fibroblasts allowed to distinguish different steps in the G1 phase progression and to define the concept of “restriction point” [41]. The progression through the G1 phase can be divided in several periods, which are different between cell types. For instance, the progression of fibroblasts throughout G1 could be divided in 4 periods: competence, entry, progression, and assembly (Figure 4). The stimulation of starved fibroblasts by PDGF promotes progression in early G1 until the restriction point C, defining the so-called competence, but fails to allow progression in mid- and late G1 [42–45]. Then the progression in late G1 and S phase can be achieved by subsequent stimulation with EGF or insulin [46, 47]. However, in absence of essential amino acids, cells arrest in mid-G1 at a restriction point named “V.” The progression between points “C” and “V “defines the period called entry [48] while the progression between point “V” and the mitogen-dependent restriction point (point “R”) is called progression. Finally, the period beyond the mitogen-dependent restriction point and before the burst of DNA synthesis is named assembly [49]. A minimal period of stimulation is required to reach the late G1 and, beyond this point, the cell cycle is completed even after removing growth factors. This restriction point is very similar to the start point in yeast that controls the commitment to S phase [50]. It is essential to distinguish the G1 progression between cells that proliferate actively and enter G1 after completion of mitosis and cells reentering the cell cycle after a prolonged quiescence or G0. The transition from G0 to G1 is characterized by a profound modification of the expressed gene profile [2] required for metabolic adaptation to cell proliferation and resulting in a longer period of time for the cells to initiate progression in late G1 compared to the cells exiting mitosis. 

**Figure 5:**
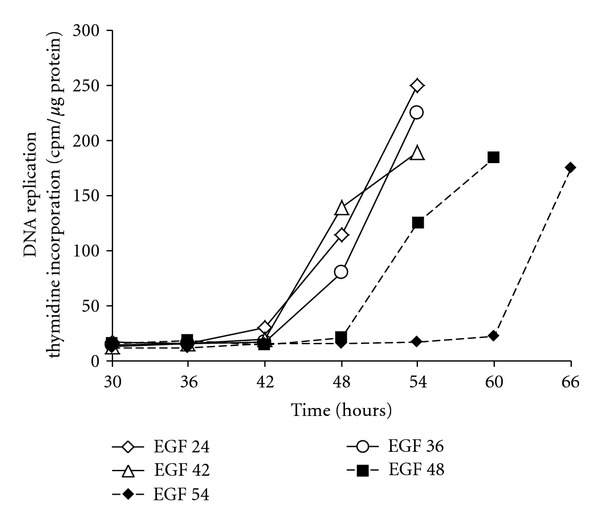
Evidencing the R point in primary rat hepatocytes. In absence of EGF rat hepatocytes do not replicate DNA. However, they sequentially express early G1 phase markers strongly suggesting a cell cycle arrest in midlate G1 phase. The mitogen-dependent restriction point was localized by performing stimulation with EGF at different time points (24, 36, 42, 48, 54 h) after seeding the hepatocytes. Then the DNA replication was monitored by measuring the incorporation of radiolabelled thymidine into the hepatocyte DNA. For stimulations between 24 and 42 h, DNA replication began between 48 and 54 h. When cells were stimulated at 48 or 54 h, DNA replication was significantly delayed demonstrating that the progression in G1 phase regardless of stimulation by EGF ended around 42 h and that the progression beyond this point required a mitogenic stimulation.

Primary cultures of rat and mouse hepatocytes were widely used to analyze hepatocyte cell cycle entry and progression through the G1 phase. Our group has shown that during cell isolation rat hepatocytes expressed immediate early protooncogenes such as c-fos and c-myc suggesting a “spontaneous” G0/G1 transition following cell-cell interaction destruction [23]. On the other hand, it had been also demonstrated that rat hepatocytes in pure culture undergo DNA replication when they were stimulated by growth factors alone [51, 52]. Thus, we hypothesized that hepatocytes were arrested in G1 phase in absence of growth factors and that by comparing unstimulated and stimulated hepatocyte it should be possible to characterize the different steps of G1 phase in hepatocytes [24]. Confirmation that collagenase perfusion of the liver induces the G0/G1 transition of quiescent normal rat hepatocytes was provided and we showed that progression in late G1 triggers hepatocyte ability to respond to growth factor alone. Importantly, demonstration that hepatocytes are able to progress from an early G1 to a mitogen-dependent restriction point (R point) located to mid-late G1 was shown (Figure 5). Indeed, in absence of growth factor and serum, hepatocytes are able to progress up to mid-late G1 phase as shown by the sequential overexpression of c-fos, c-jun, c-myc, jun D and then c-Ki-ras and p53. In addition, low levels of cyclin D1 and D2 are observed while cyclin A and Cdk1 are not expressed. Moreover, the progression towards the G1/S is strictly dependent upon the stimulation by growth factor. To further demonstrate the mitogen-dependent restriction point, we hypothesized that if the addition of EGF was performed at any time point before cells had reached the R point, the onset of DNA synthesis would not be affected. In contrast, if the addition of EGF occurred after cells had reached the R point, a delay in the onset of DNA synthesis should be observed. The hypothesis was experimentally confirmed: when addition of EGF occurred at different times but prior to 42 h after hepatocyte seeding, DNA replication took place at the same time (48–60 h) while for delayed stimulations the onset of DNA synthesis was postponed (Figure 5). A lag phase between the R point and the onset of the DNA synthesis appeared to be approximately 12–18 h. 

In this hepatocyte primary culture, Cdk2 mRNA is detectable throughout the G1 phase but significantly increased after the EGF stimulation. Cyclin A is detected at the entry of S phase and Cdk1- and Cdk2- dependent histone H1 kinase activity is mainly detected in S and M phases. Weak levels of cyclin E mRNA are found in unstimulated cultures, but levels of this mRNA greatly increased after growth factor stimulation. Surprisingly, cyclin D3 mRNAs appear to accumulate in absence of EGF stimulation whereas a drastic increase in cyclin D1 expression accompanies the R point overcrossing. The cyclin D1 mRNA accumulation correlates with the R point onset and the cyclin D1 protein is detected 10–15 h later. In accordance with these observations, accumulation of cyclin D1 is also detected when the hepatocytes are stimulated by HGF [53]. Importantly, if progression beyond the restriction is delayed by late EGF stimulation, cyclin D1 induction is postponed accordingly demonstrating that cyclin D1 induction is essential for cell cycle progression at the mitogen-dependent restriction point.

The question arises whether this restriction point existed* in vivo*. Nicely, a growth factor dependency in mid-late G1 phase of proliferating rat hepatocytes* in vivo* was also observed [54]. To reach that conclusion, we first analyzed the expression of cyclin D1 during liver regeneration and showed its induction at 12 h after hepatectomy, which is a time coinciding with the 2/3 of G1 progression as previously shown in primary culture of rat hepatocytes. We next isolated rat hepatocytes isolated 5, 7, 9, 12, or 15 h after PH and showed that only those isolated from 12–15 h regenerating livers were able to replicate DNA without growth factor stimulation. Moreover, intravenous administration of a MEK inhibitor (PD98059) *in vivo*, before MEK activation at 10.5 h post-PH, was able to inhibit cyclin D1 mRNA accumulation and hepatocyte DNA replication demonstrating that MEK/ERK signaling pathway was involved in cyclin D1 induction and R point overcrossing. To the best of our knowledge, these data provide the unique evidence that the mitogen-dependent restriction point identified in cultured hepatocytes exists *in vivo* in whole organs and animals. These results were strengthened by Albrecht's observations showing that transient enforced expression of cyclin D1 in hepatocytes stimulates assembly of active cyclin D1/cdk4 complexes, robust hepatocyte proliferation, and liver growth in rat liver [55]. However, in this *in vivo* model, after several days, hepatocyte proliferation is inhibited despite the persistence of high levels of cyclin D1 and cyclin E, suggesting that antiproliferative response related to marked upregulation of p21^Cip1^ represses cyclin D1/cdk4- and cyclin E/cdk2-dependent kinase activities. More recently, using mice carrying a floxed *EGFR* allele to inactive the EGF receptor, Natarajan et al. [56] observed delayed liver regeneration characterized by defective G1/S phase entry, reduced cyclin D1 expression followed by moderate Cdk2 and Cdk1 expression. In parallel, these authors reported an increased mortality after PH associated to high levels of TNF*α* in the serum. They also suggested that soluble TNF*α*, which is a priming agent for hepatocytes, was produced at high levels by liver cells to compensate cell cycle arrest with a subsequent induction of cell death in absence of proliferation.

Similar studies were performed in many other cell models leading to the conclusion that in all cell types the G1 phase could be divided in subphases corresponding to major steps in the metabolic adaptation required for cells to replicate DNA and divide. However, for each cell types, specific growth factors and signaling pathways are involved. Among the soluble factors inducing proliferation, the “priming” factors promote in early G1 while combination of cytokines and growth factors stimulates progression in late G1 and the G1/S transition. Then, following binding to their receptors, priming and growth factors activate multiple phosphorylation events involving multiple protein kinases especially the MAPKinase pathways [57, 58]. Moreover, multiple crosstalks between these pathways exist and lead ultimately to the activation of transcription factors that sequentially trigger induction of cell cycle regulators such as the cyclins and Cdks. 

## 4. The Cell Cycle Is Regulated through the Sequential Activation of Cdk/Cyclin Complexes

Progression of eukaryotic cells through the cell cycle is regulated by the sequential formation, activation, and subsequent inactivation of structurally related serine/threonine protein kinases, the cyclin-dependent kinase or Cdks. In mammalian cells at least 20 Cdks, 5 Cdk-like protein kinases [3], and more than 30 cyclins have been identified which form multiple Cdk/cyclin complexes controlling the cell cycle progression [59] and regulating gene transcription and RNA processing [60]. Cdks become active upon binding to their regulatory and periodically expressed subunits, namely, the cyclins. Timing of activation of these complexes is determined by a variety of mechanisms including transcriptional regulation, formation of complexes between Cdks, cyclins and other regulatory partners such as Cdk inhibitors (Cdki). In addition, phosphorylation, subcellular localization, and selective proteolysis regulate the catalytic activity of these complexes.

**Figure 6:**
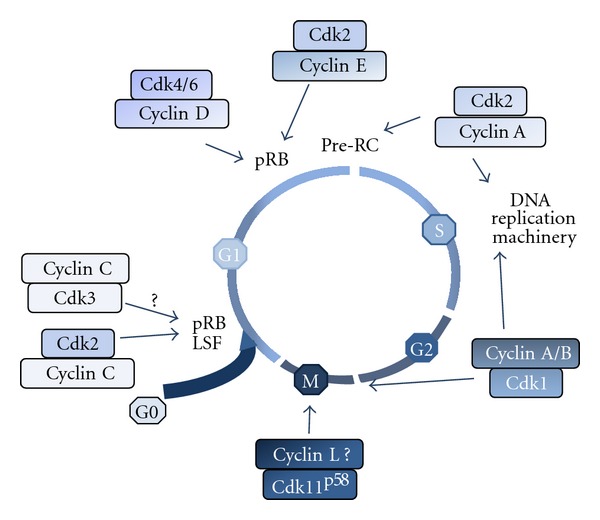
Sequential activation of Cdk/cyclin complexes throughout the cell cycle.

For many years, the G0/G1 transition and progression in early G1 phase was thought to occur in a Cdk/cyclin independent manner. Following stimulation by priming factors, immediate early genes are induced at a transcriptional level by preexisting latent transcription factors such as NF-*κ*B [61]. While cells leave quiescence to enter G1, the phosphorylation level of pocket protein family members varies [62] and inactivation of pRb is shown sufficient to induce G0/G1 transition in quiescent cells [63]. Ren and Rollins postulated that hypophosphorylated or unphosphorylated pRb present in glioblastoma T98 G0-arrested cells may be phosphorylated by Cdk3/cyclin C complexes to promote entry into G1 phase [64]. However, most cells lack functional Cdk3 and no conclusive data on the ubiquitous role of Cdk3/cyclin C complex at the G0/G1 transition have been drawn. More recently, it was reported that Cdk2 interacts with cyclin C in early G1 [65, 66] to phosphorylate the transcription factor LSF (late simian virus 40 factor) [67]. Phosphorylation of LSF on serine 291 by the MEK/extracellular signal-regulated kinase (ERK) signaling pathway upon stimulation by growth factors [58, 68, 69] in mid-late G1 phase is essential for the G1/S transition since phospho(S291)-LSF controls the transcriptional activation of the thymidylate synthase (*Tyms*) [70]. In contrast, phosphorylation of LSF on serine 309 inhibits LSF transactivation suggesting the required LSF shutdown in early G1 and its reactivation in late G1 mediated by Cdk/cyclin complexes and ERK, respectively [65]. This work appears important because it suggests a possible involvement of Cdk/cyclin complexes in early G1 and identifies LSF as the second known phosphorylation substrates of Cdk/cyclin complexes, in addition to pRb, during progression from quiescence to late G1 phase (Figure 6).

The signaling pathways essential for the subsequent progression in late G1 are much more documented and clearly involve the Cdk/cyclin complexes [71]. The transition from mid- to late G1 phase is regulated by sequential phosphorylation events of members of the pocket protein family including the retinoblastoma protein (pRb), p107, and p130 [62] by Cdk/cyclin complexes [4, 72]. In mid-G1, the hypophosphorylated pRb is bound to the transcription factor E2F family members thereby preventing active transcription of E2F-regulated genes. The negative regulation of E2F transcription factors mediated by pRb occurs through a conformation structure that prevents E2F's transactivation domain to be active and probably also by recruiting chromatin-modifying enzymes repressing transcription [73]. Upon stimulation by growth factors, D-type cyclins are upregulated [74] and associate with Cdk4 and/or Cdk6 to form active complexes [75, 76] that partially phosphorylate pRb and/or actively phosphorylate a fraction of pRb [72]. In late G1, formation of Cdk2/cyclin E complex triggers additional phosphorylation of pRb to generate the hyperphosphorylated form of pRb (Figure 6) that loses the ability to repress the transactivation domain of E2F's factors [77]. Consequently, the release of E2F proteins promotes transcription of a large set of genes required for the progression in late G1 including Cdk2 and cyclin E [78, 79], S phase entry [80–82] and centrosome duplication [83]. In parallel, Cdk2 phosphorylates the nuclear protein ataxia-telangiectasia implicated in the transcription of histones [84] and the nucleophosmin/B23 regulating centrosome duplication [85]. At this stage of the cell cycle progression cells have committed to DNA replication. Thus, turning on the E2F-dependent transcription coincides with the progression beyond the mitogen-dependent restriction point identified by Pardee and coworkers [49] before the discovery of Cdk/cyclin complexes. 

Importantly, single or combined genetic alterations in mice of Cdk4/6-Cyclin D, Cdk2-Cyclin E, p27Kip1, and Rb do not affect early embryogenesis highlighting multiple compensatory mechanisms and overlapping role of these genes introducing the notion of redundancy and flexibility of the cyclin/cdks [86, 87] The analysis of the cell cycle in MEFs derived from these knockout mice indicated compensatory mechanism between positive and negative regulators at the G1/S transition and highlighted a complex network regulating the expression and activation of these cell cycle regulators in the progression from G1 to S phase. For instance, mouse embryos lacking all interphase Cdks (Cdk2, Cdk3, Cdk4, and Cdk6) undergo organogenesis and develop up to midgestation. In these embryos, Cdk1 binds to all cyclins, resulting in the phosphorylation of the retinoblastoma protein pRb and the expression of genes that are regulated by E2F transcription factors [88]. Interestingly, cyclin A ablation in fibroblasts did not affect proliferation but led to prolonged expression of cyclin E whereas its expression is essential for cell cycle progression of hematopoietic cells and embryonic stem cells [89]. Therefore, compensatory mechanisms and overlapping role of Cdks exist but vary between cell types.

The *in vivo* model of regenerating liver was used for cell cycle studies since hepatocyte progression in the cell cycle is naturally synchronous with a long lasting G1-phase. Our group and others investigated Cdk2 and Cdk1 expression and activity as well as cyclin A, B, E, and D1 expression during liver regeneration [31, 90–92]. Although Cdk2 is constantly expressed, Cdk1 is completely absent in resting hepatocytes and remains undetectable up to 20 h after PH a time corresponding to late G1 phase and G1/S transition. In quiescent hepatocytes, Jaumot et al. [93] demonstrated that cyclin D3 and Cdk4 were localized in cytoplasm whereas cyclin D1 was nuclear. Low amounts of cyclin E are found in the cytoplasm [94]. Around 13 h after PH, cyclins D3 and Cdk4 translocate in the nucleus and significant amounts of cyclin D1/Cdk4 and cyclin D3/Cdk4 complexes are formed but remain inactive whereas at 24 h they are fully active. At 13 and 24 h, cyclin E is detected in both cytoplasm and nuclei. Then, the activity of Cdk4 decreases at 28 h when cyclin D1 translocates to the nuclear matrix and the levels of cyclin D3 diminishes. Similarly, the inactivation of Cdk2 at 28 h is associated with a strong decrease in Cdk2 in the nuclear fraction and a decrease of cyclin E located in the nuclei. During this period, very low amounts of cyclin A are detected in the nuclear fraction at 13 h after PH while following its strong induction in S phase, cyclin A is present in both cytoplasm and nuclei at 24 and 28 h. Therefore, the specific nuclear localization of the complexes is associated with their activity in liver regeneration. The maximal activity of Cdk2 detected at 24 h comes from cyclin E/Cdk2 and cyclin A/Cdk2 complexes whereas the activity at 28 h is mainly attributable to the Cdk2/cyclin A heterodimer. However, the activity of Cdk2 rapidly decreases after the peak of DNA synthesis at 24 h.

The Cdk inhibitors (Cdki's) are involved in cell cycle regulation following antagonist mitogenic and antimitogenic signals [95, 96]. Two families of Cdki's were described: the Ink4 family (p16^Ink4a^, p15^Ink4b^, p18^Ink4c^ and p19^Ink4d^), which specifically bind Cdk4 and its homologue Cdk6 and the Cip/Kip family (p21^Cip^, p27^Kip1^, p27^Kip2^), which bind and inhibit the activity of a wide range of Cdk/cyclin complexes including cyclin D/Cdk4/6, cyclin E/Cdk2, and cyclin A/Cdk2 [96]. The presence of inactive cyclin D/Cdk4 complexes during mid-G1 phase post-PH and Cyclin E/cdk2 at 28 h has led authors to investigate the modulation of Cdk kinase activities during rat liver regeneration. During rat liver regeneration, p27^Kip1^ is associated with inactive cyclin D/Cdk4 complexes [93]. Furthermore, Pujol et al. [94] have demonstrated that high amounts of p27^Kip1^ bind to Cdk2/cyclin E complexes in early and mid-G1 post-PH concomitantly with low Cdk2 kinase activities. At 24 h, corresponding to the S phase, the amounts of p27^Kip1^ associated to Cdk2/cyclin E decrease strongly while Cdk2 activity is maximal. Conversely, the amount of p21^Cip^ associated with these complexes is low during the first 13 h and subsequently increases. At 24 h low levels of both inhibitors associated with the complexes are detected, but increase in p21^Cip1^ and p27^Kip1^ proteins associated with Cdk2/cyclin A is observed at 28 h after the peak of hepatocyte DNA synthesis. Albrecht et al. [97, 98] confirmed these data and showed that expression of p21^Cip1^ is induced during the prereplicative phase and is maximal after the peak of hepatocyte DNA synthesis in mice. In contrast, p27^Kip1^ is present in quiescent liver and slightly induced after PH. Immunodepletion experiments suggested that p27^Kip1^ plays a role in downregulating Cdk2 activity before and after the peak of DNA replication. Interestingly, study of liver regeneration in mice lacking p21^Cip1^ indicated a marked acceleration of hepatocyte progression into the cell cycle. DNA synthesis, upregulation of cyclin A and PCNA, induction of cyclin D1- and Cdk2-associated kinase activities, and appearance of the hyperphosphorylated retinoblastoma protein (pRb) occur earlier in the p21^Cip1^ knockout mice. These results demonstrate the role of p21^Cip1^ in the regulation of the hepatocyte progression through G1 phase* in vivo*. Unexpectedly and again in contrast with the current model of mammalian cell cycle regulation, we observed that Cdk1 accumulates in S, G2, and M phase, in proliferating hepatocytes and is active during both S and M phases while one peak of Cdk2 activity is detected in S phase only [90]. 

## 5. Involvement of Cdk1 during the S Phase and G2/M Transition

In eukaryotic cells, chromosomal DNA replication is ensured through periodic and tightly controlled assembly and disassembly of prereplication complexes (pre-RC) loaded on DNA replication origins [99, 100]. In mid-late G1, the Origin Recognition Complex (ORC) containing several subunits associated to the proteins CDC6 and Cdt1 is responsible for loading a replicative helicase and the minichromosome maintenance (MCM) 2–7 subunits to form the pre-RC [100]. Interestingly, loading of the pre-RC components occurs in a low Cdk activity period [101] while at the onset of DNA synthesis the increasing Cdk-dependent kinase activities trigger the MCM complex to initiate replication and the degradation of Cdt1 to prevent reassembly of additional pre-RC [102–104]. The induction of MCM7 and the formation of the pre-RC thus occur in a very narrow period of time since in S phase, ORC1 and Cdt1 are degraded through several mechanisms including the phosphorylation by Cdks and downstream ubiquitination by SCFSkp2 ubiquitin Ligase [100, 105]. These well-documented mechanisms clearly point out the crucial role of Cdk/cyclin complexes in the regulation of pre-RCs formation. Similarly, pre-RC are activated by phosphorylations involving the protein kinase Cdc7 and the Cdk2/cyclin E complex which trigger the recruitment of Cdc45 [106], a crucial docking factor for DNA helicase and polymerases. During S phase, the heterodimer Cdk2/cyclin A also contributes to DNA replication [107–109] by phosphorylating components of the replication machinery including the Proliferating Cell Nuclear Antigen (PCNA) and DNA polymerases. The activity of Cdk2 is thus tightly associated with the entry into and progression in S phase (Figure 6). Following mitosis, daughter cells receive a single centrosome, which, like DNA, must duplicate prior mitosis. In early S phase, centriole duplication begins and by the late G2 two mature centrosomes have been generated to ensure proper chromosome segregation [83]. Duplication of centrioles is in part regulated through the G1 phase Cdk/cyclin-dependent pRb pathway [110], and there is a large body of evidence for the Cdk2/cyclin E involvement in the activation by phosphorylation of crucial regulators of centriole duplication [83]. 

The activity of Cdk1 associated with both A- and B-type cyclins is required for entry and progression through M phase in all eukaryotic cells [111]. The activity of the Cdk1/cyclin B complex, which was the first cyclin-dependent kinase activity detected in sea urchin and in *Xenopus* [112, 113], rapidly appeared to be a master regulator of the G2/M transition, in all eukaryotic cells [111] including in humans cells [114]. Recently, the Cdk11^p58^ protein kinase was also shown to be essential for mitosis [115, 116] most likely associated to the cyclin L's [117]. Because the kinase activities of Cdk2 and Cdk1 were mainly detected in G1/S and G2/M transitions respectively, they were thought to function independently at these two distinct periods without functional redundancy [106, 118]. 

This model of cell cycle control has first been challenged by the finding that some cancer cells proliferate despite Cdk2 inhibition [119]. Independently, there was a demonstration that knockout mice for Cdk2 as well as for E-type Cyclins are viable and that the cell cycle of cultured Cdk2^−/−^ mouse embryonic fibroblasts (MEFs) did not show major alterations [120–122]. In addition, in the hippocampus of Cdk2^−/−^ mouse, the proliferation of granule neurons of the dentate gyrus which undergo continuous renewal throughout life, is not altered [123]. Similarly, hematopoiesis is not affected in Cdk2 knockout mice [124].These data indicated that Cdk2/Cyclin E complexes were dispensable for commitment to S phase. Along the same line, a Cdk1-dependent compensatory mechanism regulating S phase initiation and progression was also demonstrated in DT40 chicken cells lacking Cdk2 [125]. Together, these data have led authors to propose a revised model of the cell cycle control in which Cdk1 compensates for Cdk2 ablation by controlling the G_1_/S transition, initiation of DNA replication and centrosome duplication [118, 126]. Interestingly, it was recently demonstrated that both Cdk1 and Cdk2 were required for efficient DNA replication in *Xenopus* egg extracts [127] suggesting that, at least in some nongenetically modified cell types, Cdk1 could contribute to S phase initiation and/or DNA replication. This idea was reinforced by the observation that enforced expression of constitutively active Cdk1 mutant in HeLa cells results in abnormal origin firing and premature DNA replication in early S phase and that a loss of Cdk1 activity compromised activation of late origins at late S phase [103]. In this emerging picture of the cell cycle regulation, these new data probably did not profoundly affect the roles that were initially attributed to the different Cdk/cyclin complexes but rather introduce the notion of redundancy and flexibility [71, 128].

In the light of the recent findings showing compensatory involvement of Cdk1 at the G1/S transition in Cdk2 knock-out mice and our data showing that Cdk1 was observed *in vivo* and *in vitro* at the G1/S transition in hepatocytes [90, 91], we have further investigated the role of Cdk1 in normal adult rat hepatocytes in the commitment to S phase. Cdk1 is barely detectable in quiescent hepatocytes and during G1 phase but expressed at high levels in S phase while Cdk2 is constantly expressed (Figure 2). Both Cdk1 and Cdk2, associated with cyclins A and/or B, are activated during DNA replication in regenerating rat hepatocytes [33]. We demonstrated that Cdk1 activity is twice higher than Cdk2 activity during S phase in hepatocytes. Then, knock-down experiments of Cdk1 and/or Cdk2 were performed in isolated hepatocytes and human foreskin fibroblasts (HFFs) which express high and low Cdk1 levels during S phase, respectively. SiRNA-mediated repression of Cdk1 and Cdk2 significantly decreased DNA replication in hepatocytes. In HFFs, repression of Cdk2 significantly reduced the DNA synthesis while repression of Cdk1 had no effect on the rate of DNA replication but, as expected, reduced the mitotic index. In hepatocyte, the activation of Cdk1 in early S phase is further demonstrated by showing that hepatocytes arrested after G1/S transition prior to DNA replication by the iron chelator O-Trensox express fully active Cdk1 and Cdk2 [33]. Moreover, the decrease in DNA replication after knocking-down Cdk1 or Cdk2 silencing is not due to impaired formation of the prereplication complex since Mcm7 is localized in the nucleus and loaded onto chromatin. In quiescent hepatocytes, MCM7 is not expressed but its expression becomes detectable immediately after the mitogenic stimulation in mid-G1, almost concomitantly with the induction of cyclin D1 and prior the Cdk-dependent kinase activity taking place in early S phase. Thus, Cdk1 may be involved in the origin firing events downstream the formation of replication complexes in hepatocytes, in agreement with a recent study suggesting that cyclin A2–Cdk1 might function as a transregulator of late origin firing in mammals or Cdk1 is required for proper timing of origin firing [103].

These data further support and extend the conclusion that Cdk1 compensates for Cdk2 gene ablation in genetically modified mice. Indeed, we have shown the involvement of Cdk1 in S phase of normal and nongenetically modified mammalian cells. More precisely, both Cdk1 and Cdk2 play a critical role in hepatocyte cell cycle. Consistent with our observation, Satyanarayana et al. [129] showed that the timing of S phase is not altered in regenerating livers of Cdk2^−/−^. Interestingly, in Cdk2^−/−^Cdk1^+/cdk2k1^ mice, in which a Cdk2 cDNA is knocked into the *Cdk1* locus, similar regenerative response and percentage of BrdU-positive cells are obtained compared to Cdk2^+/+^ mice [130]. These data indicated that Cdk2 expressed from the *Cdk1* locus is able to mimic the cell function of endogenous Cdk2 and restore normal regeneration process and that one copy of *Cdk1* is sufficient for a normal liver response after PH. In addition, Hanse et al. [131] showed that after PH most hepatocytes enter S phase in wild-type mice whereas their number is diminished significantly in Cdk2^−/−^ mice. In addition, hepatocytes isolated from livers of cdk2^−/−^ mice respond to mitogenic stimulation but to a lower extent than hepatocytes coming from wild-type mice. Very recently, Diril et al. [132] have shown that the conditional knockout of Cdk1 in adult mouse liver does not impair S phase but results in DNA rereplication and a strong decrease in cytokinesis associated with an increase in Cdk2/cyclin A2 activity. The increase in ploidy and reduced cell number suggest that Cdk1 may not be directly involved in DNA replication but would regulate Cdk2 activity and termination of DNA replication and play a major role in mitosis. 

Altogether, these results strengthened the conclusion that physiological hepatocyte proliferation is dependent on both Cdk1 and Cdk2. While Cdk1/cyclin E complexes are not detected in normal hepatocytes, Cdk1, cyclin A, and unexpectedly cyclin B1 are localized in the nucleus of replicating cells hepatocytes and form active complexes during S phase in regenerating hepatocytes. In most mammalian cells, Cdk1/Cyclin B1 complexes localize in the cytoplasm during G2 phase [133] and are activated through a positive feedback [134] to phosphorylate cytoplasmic substrates. Then the translocation to the nucleus triggers the breakdown of nuclear envelops and mitosis. The absolute requirement of cytosolic cyclin B1 during initiation of mitosis has been proposed; however, it has also been postulated that relocating cyclin B1 to the nucleus in S phase might compromise entry into mitosis [135]. This would explain why the accumulation of nuclear Cdk1/cyclin B1 complexes during DNA replication does not trigger premature mitosis in hepatocytes. Moreover, P-Tyr15 Cdk1 found in replicating hepatocytes and known to be an inactive form of Cdk1 could also participate to this control. Noteworthy, Cdk1 is active in all hepatocytes regardless of their ploidy status, excluding a peculiar regulation or role of Cdk1 related to the tetraploidy observed in half of adult hepatocytes in rat. In addition, several data highlight the role of Cdk2 in hepatocyte progression and survival following acute mitogenic stimulation [131]. Moreover, the role of Cdk2 in proper DNA repair was reported [136] and strongly suggested that Cdk2 could be a sensor able to distinguish between moderate and extensive DNA damage to promote either survival or apoptosis. Several studies have reported that Cdk1 associated with cyclin A2 or cyclin B1 was active during S phase in proliferating hepatocytes. These reports are in disagreement with numerous studies demonstrating the activation and nuclear import of Cdk1 and cyclin B1 at the G2/M transition in most cell types. Further experiments are required to address whether Cdk1 and cyclin B1 exhibit a specific pattern of expression and activation during the cell cycle of the hepatocytes and to determine their role during S phase.

## 6. Extracellular Matrix Remodeling and Cdk2 Regulate Cdk1 Expression and Activation

In normal liver, adult hepatocytes quiescent and normally do not undergo cell division but keep the ability to proliferate in response to toxic injury and infection. In regenerating liver, most of the hepatocytes undergo cell division while maintaining their metabolic function and tissue architecture. This process involved a multitude of cellular processes including at early stage acute-phase reaction [12], induction of proangiogenic signals [137], and an important extracellular matrix (ECM) breakdown and remodeling [138] leading to transient changes in the liver architecture. Connective tissue is found around the portal triads whereas reticular fibers and small amounts of basement membrane are present between the sinusoid endothelial cells and the hepatocytes. In the portal areas, mainly type I, III and V collagens are found while type IV collagen, laminin, entactin, and nidogen form the basement membrane along the sinusoids. Fibronectin is also present in the space of Disse [139].

Some proteins involved in the structural integrity of the liver are also required for normal regeneration. For example, deficiencies in connexin-32, a gap-junction protein [140], and keratin-8, an intermediate filament forming protein [141], lead to extended liver damage after partial hepatectomy. Connexin-32 is also required for normal mitosis by mediating cellular connections during cell division. Loss of proteases also results in prolonged liver injury. Mice lacking genes encoding the serine proteases urokinase-type plasminogen activator (uPA) and tissue-type plasminogen activator (tPA) exhibit delayed regeneration whereas the deficiency of the plasminogen inhibitors leads to accelerated liver regeneration [142, 143]. Interestingly, injection or increased expression of collagenase in intact liver, associated with HGF or TGF*α*, induces hepatocyte proliferation, suggesting that ECM degradation could contribute to hepatocyte priming [144]. Conversely, Issa et al. [145] observed that failure in collagen-I degradation in mouse liver inhibits the hepatocyte proliferation response. In rat, activation of plasminogen to plasmin begins shortly after PH and stays pronounced until 3–6 h. Successive inductions of mRNA levels of the metalloproteinases (MMP)-9, MMP-2, MMP-13, MMP-14, MMP-24, involved in matrix remodeling in both normal and pathological processes, are observed in mouse. Moreover, inhibitors of metalloproteinases (TIMP)-3, TIMP-4, TIMP-1 are also upregulated. In particular, TIMP-1 expression is induced prior the onset of DNA synthesis in rat and mouse models [146, 147]. After PH, its activation is linked to the hepatocyte cell cycle since experiments based on gain of TIMP-1 function in transgenic mice result in delayed cell cycle progression whereas loss of function in knockout mice accelerates liver regeneration [147]. Activation of MMP-9 after PH, mediated by plasmin or by plasmin-activated MMP-3, is followed by activation of pro-MMP-2 in MMP-2 probably by the membrane-type 1 MMP. In early phases of the liver regeneration, MMP-9 is located in the immediate periportal hepatocytes, then, its localization extends rapidly throughout the lobule before decreasing at 72 h post-PH. In the meantime, MMP-2 expression enhances in the hepatocytes at 24 and 48 h after hepatectomy [148]. Interestingly, migration of the MMP's staining pattern correlates with the gradual hepatocyte progression into the cell cycle from the periportal to the pericentral areas. This could be related to an important regulatory mechanism for controlling cell proliferation through the proteolytic maturation and/or liberation of priming and growth factors during ECM remodeling. In accordance, mature HGF production is delayed by 12 h in the uPA^−/−^ mice along with a delayed DNA synthesis. Loss of uPA results in decreased plasmin levels responsible for activating MMP that in turn digest the ECM and allow release of activated growth factors like HGF from ECM [149]. Deletion of the mouse gene *Timp3* results in the increase in TNF-*α* converting enzyme activity (TACE), constitutive release of TNF*α* and activation of TNF*α*-dependent signaling in the liver. In mice lacking *Timp3* gene, cyclin D1 and PCNA expression as well as hepatocyte division occur earlier than in wild-type mice with a shortened cell cycle. However, these mice succumbed of liver failure by a TNF*α*-signaling-dependent cell death demonstrating also the importance of TIMP-3 in controlling TNF*α* bioavailability [150].

**Figure 7:**
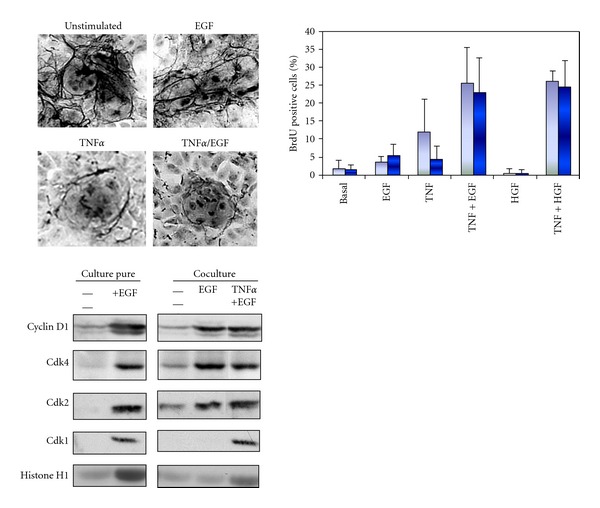
Upper left panel: detection of the extracellular matrix using the reticulin staining in unstimulated and EGF, TNF*α* or TNF*α* + EGF stimulated rat hepatocytes. Upper right panel: detection of BrdU positive hepatocytes in cocultured hepatocytes 3 days after stimulation with EGF, TNF*α*, HGF or combination of TNF*α* and EGF or HGF. Basal condition: unstimulated cells. Light blue: without fetal calf serum, dark blue: with fetal calf serum. Lower panel: expression of Cdks and cyclins in cultured rat hepatocytes. Cyclin D1, Cdk4, Cdk2, and Cdk1 were analyzed by western blotting. In addition, kinase activities of Cdk1 and Cdk2 were measured using histone H1 as a substrate.

Studies performed *in vitro* have shown that TNF*α* induces MMP-9 expression in mouse hepatocytes [151] and that MMP-9 transcription involves activation of NF-*κ*B pathway [152]. Cytokine-specific regulation of MMP/TIMP expression in hepatic stellate cells also suggests that the initial matrix degradation during liver injury might be enhanced by TNF*α*, while diminished matrix degradation during chronic tissue injury might be due to the action of TGF-*β*1 through TIMP induction [153]. Together, these studies clearly demonstrated the importance in matrix remodeling to promote proliferation of adult hepatocytes. This conclusion is reinforced by the observation that normal rat hepatocytes plated on denatured collagen I are able to proliferate following stimulation by EGF while they do not respond to this growth factor when plated on native collagen I gel [154], collagen sandwich [155], or matrigel [156]. To further address the role of the extracellular matrix degradation to promote cell cycle entry and progression of differentiated adult hepatocytes following stimulation by mitogenic signals, the primary pure culture of hepatocytes did not appear as a pertinent model since hepatocytes progress regardless of priming factors in this model. In addition, we had previously shown that very low amounts of ECM were synthesized in pure culture. We therefore used quiescent adult rat hepatocytes in coculture with liver biliary epithelial cells (Figure 7). Indeed, as mentioned above, hepatocytes in cocultures are stably differentiated for several weeks and capable of extracellular matrix deposition. This ECM located around the hepatocyte cords contains high amounts of type III, I collagens and fibronectin as *in vivo* [36]. Moreover, cytoskeleton organization of hepatocytes is similar in coculture and *in vivo* with a localization of the cytokeratins beneath of the plasma membrane [35, 157] and bile canaliculi structures present between the hepatocytes are also functional. Using this coculture system, we established new conditions allowing rat hepatocytes to undergo several proliferation waves (Figure 7) without loss of differentiation in presence of the priming cytokine, TNF*α*, and growth factors, such as HGF, EGF as observed *in vivo *during liver regeneration [39]. 

This model of controlled induction of hepatocyte proliferation has been crucial to define whether the signaling mechanisms induced by TNF*α* could be linked to ECM remodeling (Figure 7). The quantification of ECM deposition detected using reticulin staining on cells stimulated by EGF alone, TNF*α*/EGF, or successively by EGF and then TNF*α* revealed several crucial data: (1) ECM is very abundant in both unstimulated and EGF-treated cells, (2) in TNF*α*/EGF-treated cocultures, ECM deposition is very sparse and most fibers disappear within colonies of proliferating hepatocytes, (3) TNF*α* stimulation, before or after EGF exposure, induces ECM degradation, (4) during prolonged TNF*α*/EGF stimulation, DNA synthesis decreases concomitantly with new ECM deposition. In addition, the phenanthroline, a specific inhibitor of MMP activities reduces the TNF*α*-mediated ECM degradation resulting in the decrease in DNA replication. Additional experiments further demonstrated that the ECM degradation was due to the NF-*κ*B-mediated induction of MMP-9 expression by TNF*α* [39]. Thus, ECM proteolysis controlled by TNF*α* via activation of the NF-*κ*B pathway and induction of MMP-9 is necessary for S phase entry in hepatocytes. This ECM remodeling signal is also required for initiating any subsequent hepatocyte division wave in presence of mitogen [39]. These observations have been confirmed by Olle and coworkers using MMP-9^−/−^ mice [158]. Indeed, in these animals hepatic regenerative response is delayed compared with wild-type control animals. Moreover, they express significantly less HGF and TNF*α* at day 2 post-PH corresponding to hepatocyte DNA synthesis in mice [158]. In addition, in hepatoma cells, TNF*α* stimulates DNA replication by causing release of TGF*α* into the culture medium through the metalloproteinase disintegrin TACE. Then, TGF*α* activates EGFR and multiple downstream intracellular signaling cascades required for DNA replication [159]. 

Using both pure culture of hepatocytes and the coculture model, we compared expression of cell cycle markers to further investigate the molecular pathways involved in the progression in late G1 phase. In unstimulated cocultures, cyclin D1 and Cdk2 are barely detectable (Figure 7). This pattern of expression, similar to that observed in unstimulated primary pure cultures of hepatocytes, suggested that they are blocked in G1 upstream the mitogen restriction point. Unexpectedly, although no BrdU-positive hepatocytes are detected in EGF-stimulated co-cultures, cyclin D1, Cdk4, and Cdk2 accumulate in this culture condition. Interestingly, even if Cdk2 was present, no histone H1 kinase activity is detected (Figure 7). Therefore, EGF alone promotes the progression beyond the mitogen restriction point in late G1 although cells arrest before S phase. Our results could be linked to previous reports showing that cyclin E and Cdk2 are present in cells plated on denatured collagen film, while hepatocytes plated on collagen gel do not proliferate and lack the Cdk2 activity [154]. Moreover, both Cdk2 and Cdk1 are active. We therefore point out a new cell cycle control in late G1 associated with ECM deposition and overcome by TNF*α* addition that triggers ECM remodeling and induction of MMP9. Importantly, TNF*α* stimulation following EGF exposition induces the expression of Cdk1 and the activation of both Cdk2 and Cdk1 kinase activities. Altogether, our results show that induction of Cdk1, correlating with the hepatocyte S phase entry, requires remodeling of the extracellular matrix and induction of the metalloproteinase MMP9 by TNF*α* stimulation. They also suggest that catalytic activation of Cdk1 may be regulated by Cdk2 kinase activity. This led us to draw the conclusion that Cdk2 and Cdk1 would exhibit a sequential catalytic activation under the control of extracellular signals including cytokines, growth factors as well as extracellular matrix remodeling. TNF*α*–mediated ECM remodeling is necessary for Cdk2 activity, Cdk1 expression, G1/S transition, and completion of the cell cycle of hepatocytes in co-cultures.

## 7. Conclusion

Altogether, our laboratory and others have demonstrated the concomitant expression and activation of both Cdk1 and Cdk2 during S phase in hepatocytes and their active contribution to the DNA replication. Finally, we show that Cdk1 expression and activation are correlated to ECM degradation via the involvement of the proinflammatory cytokine TNF*α*. We thus identified for the first time a new signaling pathway regulating Cdk1 expression at the G1/S transition upon stimulation by cytokines. The peculiar biphasic pattern of Cdk1 activity during cell cycle of normal hepatocytes and the evenly active Cdk1 and Cdk2 during S phase contrasts with most mammalian cell types in which active Cdk2 is highly predominant over other Cdks in S phase. In most cell types, the low levels of expression and activation of Cdk1 in S phase led to the conclusion that Cdk1 and Cdk2 were functionally exclusive with specific functions in G2/M and G1/S transitions, respectively. However, in absence of Cdk2, Cdk1 can fully compensate for S phase function of Cdk2 but fails to compensate for Cdk2's DNA repair functions in mammalian cells. Based on the data obtained by our laboratory and others, we hypothesize that those high levels of active Cdk1 and Cdk2 following G1/S transition could participate to cellular defense response following stress stimulus in controlling rapid DNA repair and synthesis. We also showed that Cdk1 expression and activation are correlated to ECM degradation via the involvement of the proinflammatory cytokine TNF*α*. We thus identified for the first time a new signaling pathway regulating Cdk1 expression at the G1/S transition upon stimulation by cytokines. It also further confirms the well-orchestrated regulation of liver regeneration via multiple extracellular signals and pathways. Several important questions remain unanswered. How does TNF*α* induce Cdk2 kinase activity? It could be hypothesized that low levels of the Cdk inhibitor p27^Kip1^ following TNF*α* stimulation favors activation of Cdk2/cyclin E and Cdk2/cyclin A kinase activities. In addition, the mechanism by which TNF*α* induces Cdk1 expression remains unclear. Does it involve a transcriptional regulation mediated by unidentified signaling pathways and transcription factors? Local remodeling of the ECM could lead to disruption of ECM-cell communications achieved by integrins. Through multiple protein-protein interactions and signaling events, they could activate various signaling cascades regulating transcriptional activities. For example, repression of integrin-linked kinase (ILK), a cell-ECM-adhesion component implicated in cell-ECM signaling via the integrins, leads to enhanced cell proliferation and hepatomegaly [160]. 

## Abbreviations

H: Hours;
LDH: Lactate deshydrogenase
EGF: Epidermal growth factor
ECM: Extracellular matrix
TGF*α*: Transforming growth factor alpha
TNF*α*: Tumor necrosis factor alpha
HGF: Hepatocyte growth factor
Cdk: Cyclin-dependent kinase.
